# Size-Dependent and Multi-Field Coupling Behavior of Layered Multiferroic Nanocomposites

**DOI:** 10.3390/ma12020260

**Published:** 2019-01-14

**Authors:** Yang Shi, Yongkun Wang

**Affiliations:** 1 School of Mechano-Electronic Engineering, Xidian University, Xi’an 710071, China; ykwang@xidian.edu.cn; 2 Research Center for Applied Mechanics, Xidian University, Xi’an 710071, China

**Keywords:** multiferroic nanocomposites, magnetoelectric effect, flexoelectricity, multi-field coupling

## Abstract

The prediction of magnetoelectric (ME) coupling in nano-scaled multiferroic composites is significant for nano-devices. In this paper, we propose a nonlinear multi-field coupling model for ME effect in layered multiferroic nanocomposites based on the surface stress model, strain gradient theory and nonlinear magneto-elastic-thermal coupling constitutive relation. With this novel model, the influence of external fields on strain gradient and flexoelectricity is discussed for the first time. Meanwhile, a comprehensive investigation on the influence of size-dependent parameters and multi-field conditions on ME performance is made. The numerical results show that ME coupling is remarkably size-dependent as the thickness of the composites reduces to nanoscale. Especially, the ME coefficient is enhanced by either surface effect or flexoelectricity. The strain gradient in composites at the nano-scale is significant and influenced by the external stimuli at different levels via the change in materials’ properties. More importantly, due to the nonlinear multi-field coupling behavior of ferromagnetic materials, appropriate compressive stress and temperature may improve the value of ME coefficient and reduce the required magnetic field. This paper provides a theoretical basis to analyze and evaluate multi-field coupling characteristics of nanostructure-based ME devices.

## 1. Introduction

Multiferroic materials possess two or more unique ferroic properties simultaneously, such as ferromagnetism, ferroelectricity and ferroelasticity [1]. A composite with ferromagnetic (FM) phase and ferroelectric (FE) phase, defined as magnetoelectric (ME) composites, is a type of multiferroic material that can achieve ME coupling via mediated strain between two phases. Many types of ME composites have been reported to enhance the ME effect, including particulate composites [2], fiber-array composites [3,4], and laminate composites [5,6,7]. In particular, layered ME composites have attracted intensive research interests due to higher coupling and reduction in charge leakage. The layered ME composites have promising applications in the novel multifunctional devices, such as gyrators, sensors, energy harvesters and microwave devices [8,9,10]. In recent years, with the increasing need for realizing miniature and integrated ME devices, layered multiferroic nanocomposites with unique superiorities have attracted much attention [11]. To better fulfill the practical requirements of multiferroic nanostructures and improve their performance, the quantitative prediction of ME behavior of these nano-scaled structures seems to be a critical issue.

It has been proved that the physical and mechanical properties of nano-materials are size-dependent [12]. For the large surface to volume ratio in nanosized materials, the influence of surface effect cannot be negligible in understanding their mechanical properties. In order to simulate the surface effect of nano-materials, it is a conventional method to employ size-dependent continuum theories. Gurtin and Murdoch [13] developed a linear surface elasticity model, in which two surface layers of zero thickness are regarded as thin elastic membranes adhered to the bulk core. Huang and Yu initially extended this surface elasticity model to incorporate the surface piezoelectricity [14]. After that, a number of models considering both surface piezoelectricity and piezomagnetic effect have been developed for investigating the nano-ME system [15,16], in which flexoelectricity induced by a strain gradient or an inhomogeneous deformation [17,18] in multiferroic composites is ignored. It is widely believed that strain gradient in materials at the macro-scale is negligible, so flexoelectricity due to the strain gradient has no value in practical applications. Thus, there are only a few research works focusing on flexoelectricity of multiferroic composites in the early days. Majdoub et al. [19] revealed that the flexoelectric effect has a remarkably size-dependent property, playing an important role in the effective piezoelectric effect and mechanical behavior of nanostructures. Since the ME coupling in multiferroic composites involved in magnetic and electric reciprocal interactions between two phases, flexoelectricity of FE materials inevitably affects the ME effect [20,21]. Considering both the electrostrictive effect in the ferroelectric phase and the magnetostrictive effect in the ferromagnetic phase, Chen and Soh [22] studied the influence of flexoelectricity effect on the nonlinear ME effect in multiferroic thin bilayer films. Zhang et al. [23] focused on the effect of flexoelectricity on the linear ME effect in a multiferroic composite bilayer, in which the influence of the polarization gradient effect and surface effect are neglected.

In addition, many FM materials (e.g., Terfenol-D and Ni) present complex multi-field coupling characteristics, which have a significant impact on the ME response of ME composites under combined stress, magnetic and thermal loadings. Previous work conducted on bulk ME composites showed that the interplay of these external fields makes the optimal design of ME devices more complicated [24,25,26,27,28,29]. As for the multiferroic composites with nano-scale thickness, the applied external stimuli (e.g., magnetic field, pre-stress and temperature) influence the magnetostriction and magnetization of FM phase, so they may have effects on strain gradient as well as the materials’ properties of FM materials. To the best of the authors’ knowledge, there is no report in the literature studying the influence of external fields on strain gradient and flexoelectricity of multiferroic nanocomposites. It is expected that ME performance of multiferroic nano-devices can be accurately evaluated by exploring size-dependent behavior and availably improved by optimization of multi-physical loadings. As such, it is urgent and necessary to establish a theoretical model to analyze the size-dependent behavior and nonlinear multi-field coupling properties of multiferroic nanocomposites.

Enlightened by the investigations mentioned above, in this paper, a nonlinear multi-field coupling model for ME effect in multiferroic nanocomposites is proposed. A nonlinear constitutive relation, theories of continuum mechanics, surface elasticity and strain gradient are combined to obtain the expression of the ME coefficient. The influence of surface effect and flexoelectricity on ME coupling is numerically studied and discussed in detail. Then the strain gradient under different external fields are evaluated for the first time. Finally, the multi-field coupling characteristics of multiferroic composites are analyzed for the improvement of ME performance.

## 2. Basic Equations for Constituent Materials

In this section, we first summarize the basic equations for constituent materials with size-dependent effects (e.g., flexoelectricity and surface effect). Based on G-M theory, when the thickness of layered multiferroic materials shrinks down to nanoscale, it is assumed that the objects consist of two parts: surface layer and bulk core. The surface layers of nano materials, which has different material properties with the bulk core, are considered as thin membranes with negligible thickness perfectly adhered to the bulk. Experiments have proved that the bulk core of some FM materials show nonlinear magneto-elastic-thermal coupling property. That is, the properties of these materials are significantly related to the external stress, magnetic and thermal loadings. The nonlinear magneto-elastic-thermal coupling constitutive relations of magnetostrictive materials is written as [30]

(1) $$\begin{array}{l} \sigma =C\varepsilon +\alpha \Delta T+\sigma (\varepsilon ,M,\Delta T)\\ H=[\frac{1}{kM}f^{-1}(\frac{M}{M_{s}})I-\frac{\lambda_{s}}{\mu_{0}M_{s}^{2}}\bar{\sigma }]M \end{array}$$

where σ and ε are the first-rank stress and strain tensors, C is the second-rank stiffness coefficient tensor, H and M are the first-rank magnetic field and magnetization tensors, I is the second-rank unit tensor, $\lambda_{s}$ and $M_{s}$ are the saturated magnetostriction and magnetization, k is relaxation factor with $\chi_{m}$ being linear magnetic susceptibility, $\mu_{0}$ is vacuum permeability, f(x) is Langevin function, and $M=\sqrt{M_{1}^{2}+M_{2}^{2}}$. The reader is referred to Reference [31] for the more detailed expressions of C, σ(ε,M,ΔT), and $\bar{\sigma }$. Since it is difficult to apply directly the nonlinear constitutive equations to build a theoretical model, we introduce variable coefficients and rewrite the constitutive equations into a linear-like form

(2) $$\begin{array}{l} \sigma_{ij}=c_{ijkl}^{m}\left(\begin{array}{ccc} \sigma_{ij}, & H_{k}, & \Delta T \end{array}\right)\varepsilon_{kl}+\alpha_{i}^{m}\Delta T-g_{kij}^{m}\left(\begin{array}{ccc} \sigma_{ij}, & H_{k}, & \Delta T \end{array}\right)H_{k}\\ B_{k}=g_{kij}^{m}\left(\begin{array}{ccc} \sigma_{ij}, & H_{k}, & \Delta T \end{array}\right)\varepsilon_{ij}+\xi_{i}^{m}\Delta T+\mu_{ki}^{m}\left(\begin{array}{ccc} \sigma_{ij}, & H_{k}, & \Delta T \end{array}\right)H_{k} \end{array}$$

where the right superscript *m* refers to piezomagnetic phase. $\xi_{i}^{m}$ is the slope of the magnetic flux density versus temperature, $c_{ijkl}^{m}$, $g_{kij}^{m}$, and $\mu_{ki}^{m}$ are the equivalent elastic coefficient, piezomagnetic coefficient and magnetic permeability constant, respectively, which are functions of magnetic field, stress and temperature and determined by

(3) $$\begin{array}{ccc} C^{m}=\frac{d\sigma^{m}}{d\varepsilon^{m}}, & g^{m}=\frac{d\sigma^{m}}{dH}, & \mu^{m}=\mu_{0}\left(I+\frac{dM}{dH}\right) \end{array}$$

For the bulk core of FE materials, piezoelectric theory [32] with considering the coupling between strain gradient and polarization is written as

(4) σij=cijklpεkl+αipΔT−eijkpEkτijk=−μijklpElDl=eijkpεjl+pipΔT+aljpEj+μijklpεjkl

where the right superscript *p* refers to piezoelectric phase. $\sigma_{ij}$ is the stress, $\tau_{ijk}$ is the higher order stress, $c_{ijkl}^{p}$, $e_{ijk}^{p}$, and $a_{lj}^{p}$ are the material constants defined in Vogit’s theory of piezoelectricity, $\mu_{ijkl}^{p}$ is flexoelectric coefficient, $\alpha_{i}^{p}$ and pip represent the thermal expansion coefficient and the pyroelectric coefficient, $E_{k}$ and $D_{l}$ are electric field and electric displacement vector.

Surface stress will be generated unavoidably on the surface layers, which may depend on surface strain, electric field and magnetic field. The surface constitutive equations of FE and FM phases can be written as

(5a) σijps=cijklpsεkls+αipsΔT−eijkpsEjsDls=eijlpsεjlps+pipsΔT+aljpsEjs

(5b) σijms=cijklmsεkls+αimsΔT−gkijmsHksBks=gkijmsεijs+ξimsΔT+μkimsHks

where the left superscript *s* refers to surface layer. σijs, $\varepsilon_{kl}^{s}$, Ejs, Dls, Hks, and Bks are the surface stress, surface strain, surface electric field, surface electric displacement vector, surface magnetic field and surface magnetic induction, respectively. cijklps, eijkps, and αips are surface material constants of FE materials, cijklms, gkijms, and μkims are surface material constants of FM materials. The material constants of the surface layers can be obtained by atomistic calculations or experiments. However, due to the heavy computations of atomistic calculations and the complicated operation conditions of the relevant experiments, the accurate values of the surface layers of most FE and FM materials are not yet available. Gurtin and Murdoch [9] concluded that the surface modules could merely be expressed as the scaled versions of their bulk counterparts ($p_{s}$), Thus, the relevant material constants within the surface layer can be evaluated by [13,33]

(6) cijkls=lscijkl,eijkps=lseijkp,aljps=lsaljp,gkijms=lsgkijm,μkims=lsμkim

in which $l_{s}$ refers to a material intrinsic length, which can be taken as the thickness of the surface layer.

## 3. Modeling of Multiferroic Nanocomposites

**Figure 1:**
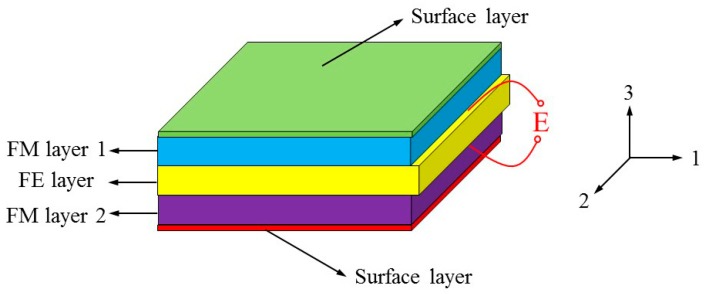
Schematic sketch of multiferroic nanocomposites consisting of ferroelectric (FE) material and different ferromagnetic (FM) materials.

The schematic diagram of the multiferroic nanostructured films consisting of FE materials and different FM materials are shown in Figure 1. The present work uses a multiferroic nanocomposite with L-T mode configuration in which the magnetostrictive material is magnetized in longitudinal (L) direction under combined ac and dc magnetic field, and the piezoelectric material is poled in transverse (T) direction [34]. The thickness of the composites and each layer are denoted as t and $t_{i}$, where the subscript $i=\begin{array}{ccc} m_{1}, & p, & m_{2} \end{array}$ represents the upper FM layer, FE layer and lower FM layer, respectively. A coordinate system with the origin at the center of each layer is assumed, in which *x*-, *y*-, and *z*-axes are along length, width, and thickness directions, respectively. Since the thickness and the width of the composites are considerably smaller than its length, we can assume that only the components of the stress and strain tensors along direction 1 are nonzero. As shown in Figure 1, two surface layers are regarded as thin elastic membranes adhered to the upper and lower surface of nanostructured film. The surface layer and the bulk core have different material properties. As for the interfaces, the existing literatures have reported two independent interface stresses: one is associated with a coherent interface in which no atomic bonds are broken, so the tangential or interior strain components are equal on both sides of the interface, while the other allows for different tangential strains at the two sides [35]. Here we are concerned with the former and ignore the thickness of interface layer, i.e., it is assumed that the interface between each two layers is perfect. The strain at the interface satisfies the following interfacial conditions.

(7a) $${\varepsilon_{1}^{p}|}_{z_{p}=\frac{t_{p}}{2}}={\varepsilon_{1}^{m_{1}}|}_{z_{m_{1}}=-\frac{t_{m_{1}}}{2}}$$

(7b) $${\varepsilon_{1}^{p}|}_{z_{p}=-\frac{t_{p}}{2}}={\varepsilon_{1}^{m_{2}}|}_{z_{m_{2}}=\frac{t_{m_{2}}}{2}}$$

Non-uniform distributed strain is induced under the applied magnetic field due to the asymmetry of configuration with different FM layers. Thus, the coupled extensional and flexural deformations appear simultaneously in the layers. According to Kirchhoff’s hypotheses, the longitudinal axial strains of each layer are given as

(8) $$\varepsilon_{1}^{i}=\varepsilon_{10}^{i}+\frac{z_{i}}{\rho }$$

where $\varepsilon_{10}^{i}$ refers to the centroidal strains at $z_{i}=0$ of each layer which is along the *x*-axes, and ρ is the curvature of composites.

Accordingly, the strain gradient components can be written as

(9) $$\varepsilon_{11,3}=\frac{\partial \varepsilon_{1}}{\partial z}=\frac{1}{\rho }$$

Considering the interfacial condition in Equation (7) and the geometrical relationship in Equation (8), it can be derived that

(10) $$\begin{array}{l} \varepsilon_{10}^{p}+\frac{t_{p}}{2\rho }=\varepsilon_{10}^{m_{1}}-\frac{t_{m_{1}}}{2\rho }\\ \varepsilon_{10}^{p}-\frac{t_{p}}{2\rho }=\varepsilon_{10}^{m_{2}}+\frac{t_{m_{2}}}{2\rho } \end{array}$$

The left of Equation (10) is the in-plane strain of piezoelectric phase at the interface, and the right is the one of FM phases. Then, combining Equations (8) and (10), the strain components are drawn as

(11) $$\begin{array}{cc} \varepsilon_{1}^{m_{1}} & =\varepsilon_{10}^{m_{1}}+\frac{z_{m_{1}}}{\rho }\\ \varepsilon_{1}^{p} & =\varepsilon_{10}^{m_{1}}-\frac{t_{p}}{2\rho }-\frac{t_{m_{1}}}{2\rho }+\frac{z_{p}}{\rho }\\ \varepsilon_{1}^{m_{2}} & =\varepsilon_{10}^{m_{1}}-\frac{t_{p}}{\rho }-\frac{t_{m_{1}}}{2\rho }-\frac{t_{m_{2}}}{2\rho }+\frac{z_{m_{2}}}{\rho } \end{array}$$

Based on the basic equations in Section 2, the following equations can be written for the strains of FM, FE and surface layers:

(12) ε1m=s11m(σij,Hk,ΔT)σ1m+βm(σij,Hk,ΔT)ΔT+q11m(σij,Hk,ΔT)H1ε1p=s11pσ1p+βpΔT+d31E3pε1ms=s11ms(σij,Hk,ΔT)σ1ms+βms(σij,Hk,ΔT)ΔT+q11ms(σij,Hk,ΔT)H1s

where $d_{31}$, $\varepsilon_{33}$, $s_{11}$, $q_{11}$ and β represent the piezoelectric coefficient, the relative dielectric constant, the equivalent compliance coefficient, the equivalent piezomagnetic coefficient and the thermal expansion coefficient respectively. These coefficients can be obtained from the material constants in Equations (2), (4) and (5) by the equations

(13) $$\begin{array}{l} s_{11}^{m}\left(\begin{array}{ccc} \sigma_{ij}, & H_{k}, & \Delta T \end{array}\right)=1/c_{11}^{m}\left(\begin{array}{ccc} \sigma_{ij}, & H_{k}, & \Delta T \end{array}\right)\\ \beta^{m}\left(\begin{array}{ccc} \sigma_{ij}, & H_{k}, & \Delta T \end{array}\right)=1/\alpha^{m}\left(\begin{array}{ccc} \sigma_{ij}, & H_{k}, & \Delta T \end{array}\right)\\ q_{11}^{m}=g_{11}^{m}\left(\begin{array}{ccc} \sigma_{ij}, & H_{k}, & \Delta T \end{array}\right)/s_{11}^{m}\left(\begin{array}{ccc} \sigma_{ij}, & H_{k}, & \Delta T \end{array}\right)\\ \begin{array}{ccc} s_{11}^{p}=1/c_{11}^{p}, & \beta^{p}=1/\alpha^{p}, & d_{31}^{p}=e_{31}^{p}/c_{11}^{p} \end{array} \end{array}$$

Substituting the strain components in Equation (11) into Equation (12), one can rewrite the relations between strain and stress components as

(14) $$\begin{array}{l} \varepsilon_{10}^{m_{1}}+\frac{z_{m_{1}}}{\rho }=s_{11}^{m_{1}}\sigma_{1}^{m_{1}}+\beta^{m_{1}}\Delta T+q_{11}^{m_{1}}H_{1}\\ \varepsilon_{10}^{m_{1}}-\frac{t_{p}}{2\rho }-\frac{t_{m_{1}}}{2\rho }+\frac{z_{p}}{\rho }=s_{11}^{p}\sigma_{1}^{p}+\beta^{p}\Delta T+d_{31}E_{3}^{p}\\ \varepsilon_{10}^{m_{1}}-\frac{t_{p}}{\rho }-\frac{t_{m_{1}}}{2\rho }-\frac{t_{m_{2}}}{2\rho }+\frac{z_{m_{2}}}{\rho }=s_{11}^{m_{2}}\sigma_{1}^{m_{2}}+\beta^{m_{2}}\Delta T+q_{11}^{m_{2}}H_{1} \end{array}$$

Since the FE layer is free of charge in sensor modes, we use open circuit condition $D_{3}^{p}=0$ and Equation (4) to obtain the induced electric field, expressed as

(15) $$E_{3}^{p}=-\frac{1}{\varepsilon_{33}}\left(d_{31}\sigma_{1}^{p}+\mu_{3113}\varepsilon_{11,3}+p^{p}\Delta T\right)$$

Hence, the stress components of each layer are derived from Equations (14) and (15)

(16a) $$\sigma_{1}^{m_{1}}=\frac{1}{s_{11}^{m_{1}}}\left[\left(\varepsilon_{10}^{m_{1}}+\frac{z_{m_{1}}}{\rho }\right)-\beta^{m_{1}}\Delta T-q_{11}^{m_{1}}H_{1}\right]$$

(16b) $$\sigma_{1}^{p}=\frac{\varepsilon_{33}}{s_{11}^{p}\varepsilon_{33}-d_{31}^{2}}\left[\left(\varepsilon_{10}^{m_{1}}-\frac{t_{p}}{2\rho }-\frac{t_{m_{1}}}{2\rho }+\frac{z_{p}}{\rho }\right)+\left(\frac{d_{31}p^{p}}{\varepsilon_{33}}-\beta^{p}\right)\Delta T+\frac{d_{31}\mu_{3113}}{\varepsilon_{33}}\frac{1}{\rho }\right]$$

(16c) $$\sigma_{1}^{m_{2}}=\frac{1}{s_{11}^{m_{2}}}\left[\left(\varepsilon_{10}^{m_{1}}-\frac{t_{p}}{\rho }-\frac{t_{m_{1}}}{2\rho }-\frac{t_{m_{2}}}{2\rho }+\frac{z_{m_{2}}}{\rho }\right)-\beta^{m_{2}}\Delta T-q_{11}^{m_{2}}H_{1}\right]$$

(16d) $$\tau_{311}^{p}=\left[\begin{array}{l} \frac{d_{31}\mu_{3113}}{s_{11}^{p}\varepsilon_{33}-d_{31}^{2}}\left(\frac{z_{p}}{\rho }-\frac{t_{m_{1}}}{2\rho }-\frac{t_{p}}{2\rho }+\frac{d_{31}\mu_{3113}}{\varepsilon_{33}}\frac{1}{\rho }\right)+\frac{\mu_{3113}^{2}}{\varepsilon_{33}}\frac{1}{\rho }\\ +\frac{d_{31}\mu_{3113}}{s_{11}^{p}\varepsilon_{33}-d_{31}^{2}}\varepsilon_{10}^{m_{1}}+\left[\frac{d_{31}}{s_{11}^{p}\varepsilon_{33}-d_{31}^{2}}\left(\frac{d_{31}p^{p}}{\varepsilon_{33}}-\beta^{p}\right)+\frac{p^{p}}{\varepsilon_{33}}\right]\mu_{3113}\Delta T \end{array}\right]$$

For a coherent surface, the surface strain and magnetic field have the same values as the corresponding quantities in the abutting bulk materials, given as

(17) ε1s=ε1|z=tm1or−tm2H1s=H1

Substituting the above expressions into the third constitutive equation of Equation (12), we obtain the surface stress as

(18) σ1m1s=1s11m1s[(ε10m1−tm12ρ)−βm1sΔT−q11m1sH1]σ1m2s=1s11m2s[(ε10m2+tp2ρ+tm12ρ+tm2ρ)−βm2sΔT−q11m2sH1]

The internal force *N* and moment *M* on the cross-section of the laminate can be obtained by integrating the stresses along the thickness of the individual layer as follows:

(19) $$\begin{array}{l} N=\sum_{i=1}^{n}\int_{z_{i-1}}^{z_{i}}\sigma \left(i\right)dz\\ M=\sum_{i=1}^{n}\int_{z_{i-1}}^{z_{i}}\left[\tau_{311}\left(i\right)+z\sigma \left(i\right)\right]dz \end{array}$$

where *n* is the number of layers of the composite; *z* is the relative coordinate of the normal direction of the middle plane.

To incorporate the surface effects of the ME nanocomposites, we assumed that two surface layers of vanishing thickness adhere perfectly to the bulk without any slipping. With free boundary conditions at the two ends of the nanocomposites, the axial forces in the bulk layers and surface stress in two surface layers must add up to zero to preserve force equilibrium. Simultaneously, to conserve moment equilibrium, the rotating moments of axial forces in the bulk layers are counteracted by resultant bending moments from the axial forces and surface stress. Thereby, the mechanical free conditions of ME nanocomposites are expressed as follows:

(20) N+σ1m1s+σ1m2s=0M=σ1m1stm12−σ1m2s(tp+tm2+tm12)−Np(tp+tm12)−Nm2(tp+tm2+tm12)

Substituting Equations (16) into Equation (19), then using the mechanical free conditions, we immediately obtain an equation set with respect to centroidal strain $\varepsilon_{10}^{m_{1}}$ and curvature radius ρ (given in Appendix A), which are solved on a computer using MATLAB (9.2.0.538062, The MathWorks, Inc., Beijing, China). Once the centroidal strain and radii of curvature are determined, the axial stress $\sigma_{1}$ can be found from Equation (16), and then the electric field can be calculated by Equation (15). Thus, the static ME voltage coefficient considering size-dependent behavior and magneto-elastic-thermal coupling characteristics is evaluated by

(21) $$\begin{array}{cc} \alpha_{E} & =\frac{1}{t_{p}}\frac{1}{H_{1}}\int_{-t_{p}/2}^{t_{p}/2}E_{3}^{p}dz\\ & =-\frac{1}{s_{11}^{p}\varepsilon_{33}-d_{31}^{2}}\frac{1}{H_{1}}\left[d_{31}\left(\varepsilon_{10}^{m_{1}}+\frac{t_{m_{1}}+t_{p}}{2\rho }\right)+s_{11}^{p}\frac{\mu_{3113}}{\rho }+\left(s_{11}^{p}p^{p}-d_{31}\beta^{p}\right)\Delta T\right] \end{array}$$

Since the amount of symbols and variables is significant, a symbols table is given in Appendix B, which may help the readers quickly look up the definitions of the symbols/variables.

## 4. Numerical Results and Discussions

**Figure 2:**
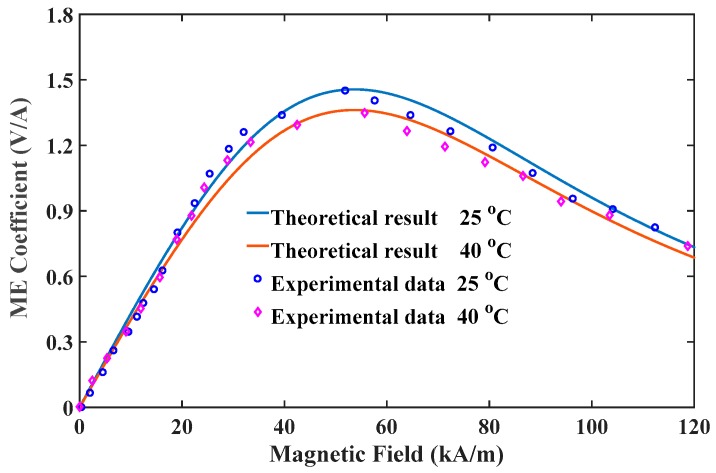
Comparison of variation of the ME coefficient at various operating temperatures.

Before performing numerical calculations, the established nonlinear multi-field coupling model should be verified by real-world data. After an extensive literature review, the authors find that the experimental data on nonlinear ME coupling of layered multiferroic nanocomposites under stress or thermal loading is relatively scarce. Fortunately, Fang and her cooperators [36] have performed an experiment on ME effect of bulk ME composites under different ambient temperatures, which provided experimental data for studying temperature effect. Although the model established above depicts the dependences of ME coefficient for asymmetric nanostructured ME composites on the size-dependent parameters, it also applies to predict nonlinear ME coupling of symmetric bulk ME composites (e.g., Terfenol-D/PZT/Terfenol-D) if surface effect and flexoelectricity are excluded. With this in mind, we choose the experimental data of Fang et al. [36] to verify our nonlinear ME effect model. Figure 2 shows the comparison of variation of the ME coefficient at various operating temperatures. In both cases, the proposed scheme is able to capture the maximum ME response and the optimum field. Besides, the predictions of temperature effect agree well with experimental data. The overall agreements indicate that the theoretical model is sufficiently accurate for characterization and design of the multiferroic composite under multi-field conditions.

**Figure 3:**
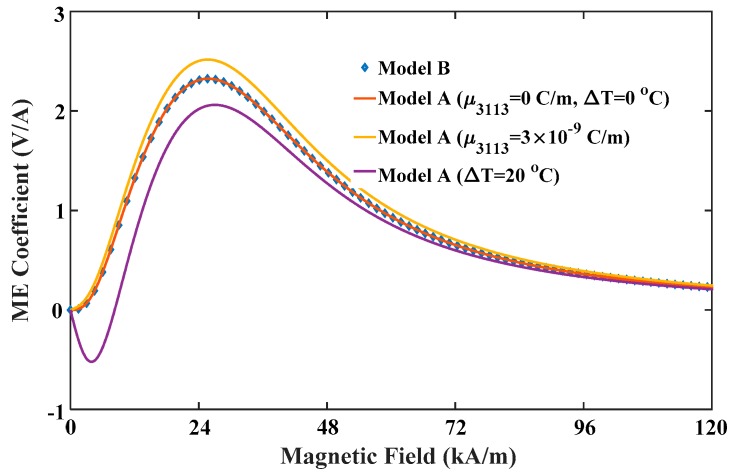
ME coefficients of Model B and Model A without flexoelectricity and temperature effect, only with flexoelectricity, and only with temperature effect.

As a case study, we choose an asymmetric Terfenol-D/PZT/Ni layered nanocomposite as the object of study. The material properties of three constituent materials are listed in Table 1 [13,37]. The volume fraction of FE material is denoted by v. It is noteworthy that the model developed here, denoted by model A, can be reduced to the one (denoted by model B) reported in our previous work [13] if flexoelectricity and temperature effect are neglected by setting the values of $\mu_{3113}$ and ΔT to zero. For the cause of comparing, two theoretical models are employed for predicting the ME coefficient of Terfenol-D/PZT/Ni nanocomposites as shown in Figure 3. When flexoelectricity and temperature effect in model A are neglected, the predicted results are in perfect agreement with model B. However, whether the flexoelectricity or temperature effect is taken into account, the ME coefficient of model A significantly deviates from the prediction of model B which only considers surface stress and magneto-elastic coupling. This comparison underscores the absolute importance of flexoelectricity and magneto-elastic-thermal coupling in predicting the ME performance of nanocomposites-based multiferroic devices. For example, on one hand, the value of the ME coefficient of Model A with flexoelectricity is larger than Model B. This is because the total electric field is a superposition of electric fields induced via piezoelectricity and flexoelectricity. In general, the piezoelectric coefficients are non-zero for only selected (piezoelectric) dielectrics, while the flexoelectric coefficients are non-zero for all dielectrics [38]. This implies that, when subjected to strain gradients, e.g., bending or inhomogeneous stretching, at least in principle, all dielectric materials are capable of producing an electric field from flexoelectricity. Therefore, an interesting use of flexoelectricity is to create some “apparently piezoelectric” materials as the FE phase of multiferroic nanocomposites even though the actual materials are not intrinsically so, which offers an opportunity for improvement of the mechanical properties of multiferroic devices [39]. On the other hand, two peak values appear in the ME coefficient curve of Model A with temperature effect. This implies that one can obtain an excellent performance of multiferroic devices under a relatively low magnetic bias. Thus, the magneto-elastic-thermal coupling is of potential importance in developing an approach to reduce the optimum *H*_dc_, or even realize an approximately zero-biased ME effect [40].

**Figure 4:**
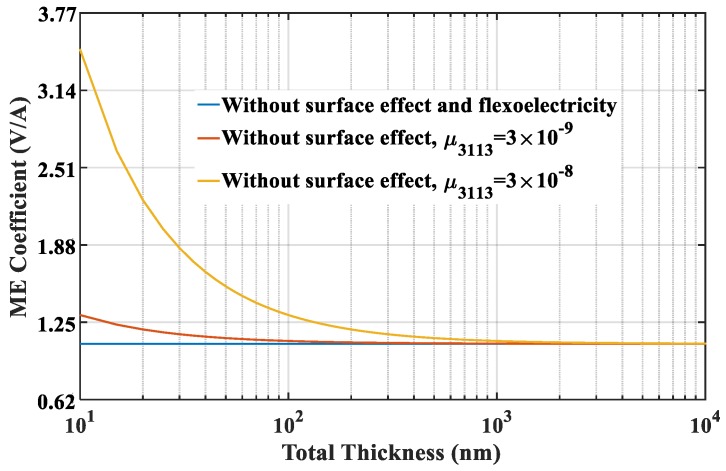
The effect of flexoelectricity of FE phase on the ME coefficient.

**Figure 5:**
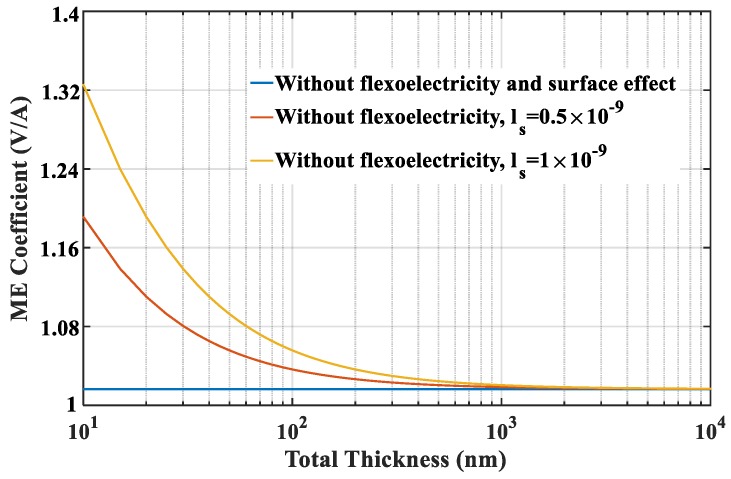
The effect of surface layer of FM phase on the ME coefficient.

Next, we focus on the size-dependent behavior of ME coupling. The applied magnetic field, pre-stress and temperature increment are fixed at 40 kA/m, −5 MPa and 20 °C, respectively. To clearly investigate the effect of flexoelectricity on the ME effect, the curves of the ME coefficients versus the total thickness of the nanocomposites for different flexoelectric coefficients are plotted in Figure 4. It can be seen that the ME coefficient without consideration of surface effect and flexoelectricity is size-independent. However, when only flexoelectricity is considered, the ME coefficient is significantly changed with the thickness. As the thickness of the nanocomposite is reduced to the nanoscale, the ME coefficient increases rapidly, which indicates that nanocomposites are advantageous for improving the ME coupling effect. A larger flexoelectric coefficient causes a more evident flexoelectricity, and thus enhances ME coefficient considerably. However, the influence of flexoelectricity gradually diminishes with increasing the thickness to macro-scale. On the basis of $\begin{array}{cc} l_{s}=1 & \mathrm{nm} \end{array}$ and $\begin{array}{cc} \mu_{3113}=3\times 10^{-8} & C/m \end{array}$, the ME coefficient for the thickness of 10 nm are three times higher than in the absence of flexoelectricity. Besides, when the thickness increases to 1 μm, the ME coefficient with flexoelectricity is reduced to a saturation value which is no longer changed with the thickness and approaches to that without flexoelectricity. Therefore, a critical thickness can be proposed by the results in Figure 4. This phenomenon suggests that flexoelectricity has a tremendous influence on the ME coupling of multiferroic composites at nanoscale. Figure 5 shows the effect of the surface layer of FM phase on the ME coefficient. The ME coefficient has a similar variation tendency with Figure 4 when only surface effect is taken into account. As shown in the figure, the presence of surface layer also leads to size-dependent ME coupling effects. The same critical value of thickness can be obtained from Figure 5, which means that surface layers can improve the ME coefficient obviously below this critical value but can be eliminated in modeling of multiferroic composites at macroscale. The curves also indicate that the ME effect will be improved by increasing the material intrinsic length.

**Figure 6:**
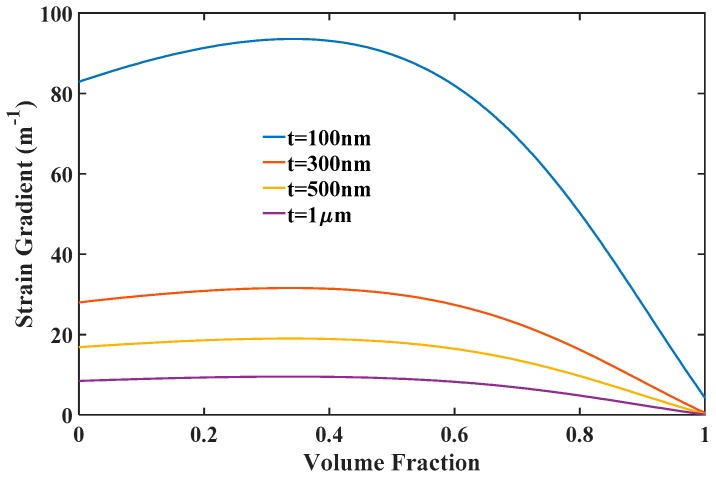
The strain gradient varied with volume fraction of the FE material.

**Figure 7:**
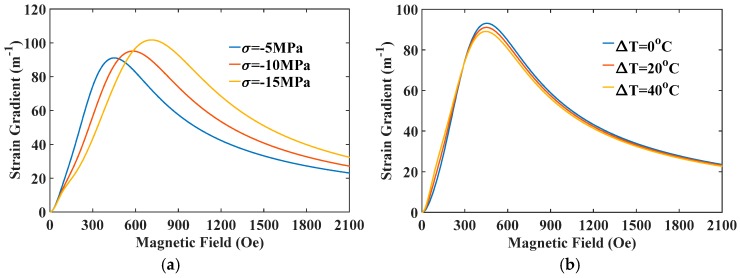
The strain gradient varied with magnetic field under different (**a**) stresses and (**b**) temperatures.

The above results show that flexoelectricity of FE materials has a positive impact on the ME effect. In the optimum design of multiferroic composites, an important key parameter resulting in a relatively large flexoelectricity is the strain gradient. The strain gradient can be evaluated from solving Equations (9) and (A-1) and plotted in Figure 6. As can be seen, the strain gradient increases rapidly as the thickness reduces from macroscale to nanoscale. That is why flexoelectricity for a given flexoelectric coefficient plays a significant role in the ME coupling behavior of multiferroic nanocomposites. In general, applied external loadings influence the material constants of FM materials greatly, so the produced strain gradient due to curvature should depend on these physical quantities. Figure 7 shows the strain gradient varied with magnetic field under different stresses and temperatures. The strain gradient first increases with increasing the applied magnetic field rapidly, reaches a maximum value, then decreases with the further increase of magnetic field. Thus, an optimal magnetic field can be obtained for the improvement of strain gradient under a given pre-stress and temperature. Figure 7a shows that the introduction of pre-stress enhances both the maximum values of strain gradient and the optimal field. It is predictable that the strain gradient induced in the multiferroic nanocomposites can be improved by decreasing compressive stress under low magnetic fields or increasing compressive stress under high magnetic fields, which is important for the experimental design and practical applications. On the contrary, it is evident in Figure 7b that a larger temperature increment can enhance strain gradient under low magnetic fields, but weakens under high magnetic fields. In any case, eliminating or reducing temperature increments will be beneficial for improving the maximum value of the strain gradient. This is not a difficult thing to achieve in practical applications. For example, semiconductor refrigeration tablets can be stuck on the surface of nanocomposites with silica gel, which can conduct heat via the Peltier effect [41], so that it can effectively reduce or even eliminate the temperature increment of FM materials. These results provide a potential possibility for optimizing the strain gradient for a given multiferroic composite structure operating under multi-field coupling conditions.

**Figure 8:**
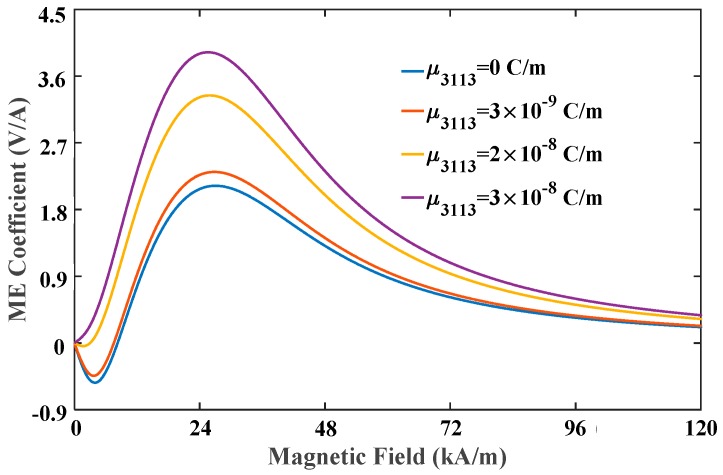
The ME coefficient varied with the applied magnetic field for different flexoelectric coefficients.

**Figure 9:**
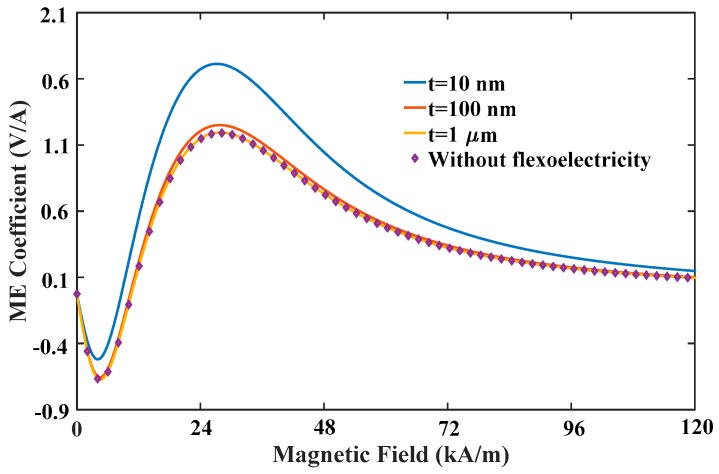
The ME coefficient varied with the applied magnetic field for different total thicknesses.

Figure 8 illustrates the variation of the ME coefficient with the applied magnetic field for different flexoelectric coefficients. Taking the case of $\begin{array}{cc} \mu_{3113}=3\times 10^{-9} & C/m \end{array}$ for instance, ME coefficient has negative value under very low field and shows a non-monotonic variation during the field sweep, leading to a dual-peak phenomenon in the curve. This novel phenomenon should be a unique feature that can be observed only for multiferroic composites using simultaneously both positive and negative magnetostrictive materials as FM phases. Taking the proposed composites, the positive magnetostrictive material Terfenol-D has positive magnetostriction that reaches saturation under high field, but the negative magnetostrictive material Ni has negative magnetostriction that reaches saturation under low field. Consequently, the piezomagnetic effect of Ni plays the dominant role and induces negative ME coefficient under low field. It should be noted that the negative ME coefficient is attributed to the phase difference between the electric field induced by negative strain and magnetic field. As the magnetic field increases, the piezomagnetic effect of Terfenol-D is enhanced and gradually takes over the leading role. The positive strain from Terfenol-D layer counteracts and reverses entirely the negative strain from Ni layer, and thus the phase difference between the electric field and magnetic field is eliminated. Therefore, the ME coefficient under high field is mainly determined by the piezomagnetic effect of Terfenol-D, which is improved by increasing field to an appropriate value. As the flexoelectric coefficient increases, the positive ME coefficient increases whereas the negative one decreases, and the first peak disappears at $\begin{array}{cc} \mu_{3113}=3\times 10^{-8} & C/m \end{array}$. This signifies that electric fields induced by flexoelectricity and positive strain have the same phase. Figure 9 illustrates the variation of the ME coefficient with the applied magnetic field for different thicknesses. As can be seen, there is an obvious increase in the ME effect with the reduction of the total thickness of composites. For example, 47% growth of the ME coefficient can be achieved when the thickness reduces from 1 μm to 10 nm. In addition, when the thickness is 1 μm, the ME coefficient with flexoelectricity is almost the same as without flexoelectricity. These results reconfirm the enhancing ME effect in multiferroic nanocomposite bilayers via flexoelectricity.

**Figure 10:**
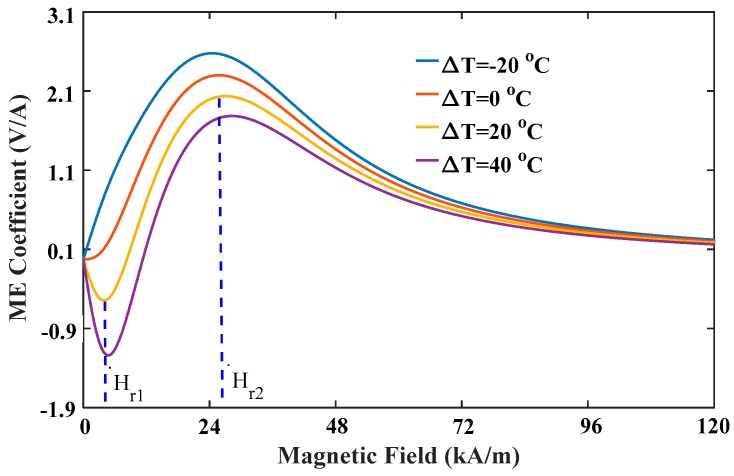
The ME coefficient varied with magnetic field under different temperatures.

**Figure 11:**
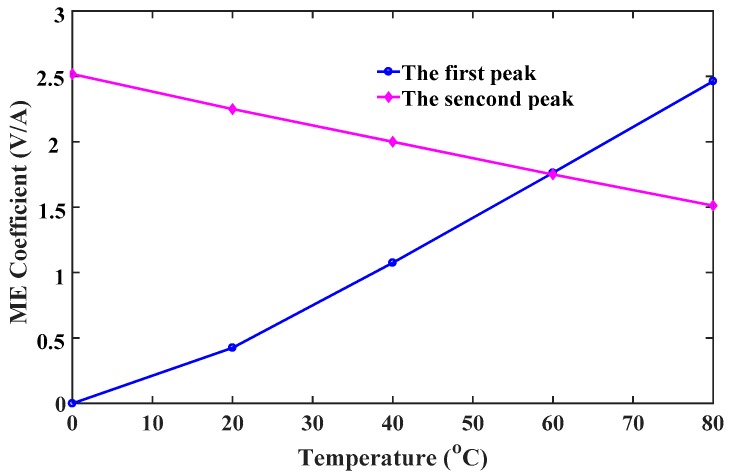
The peak values of the ME coefficient varied with temperature.

**Figure 12:**
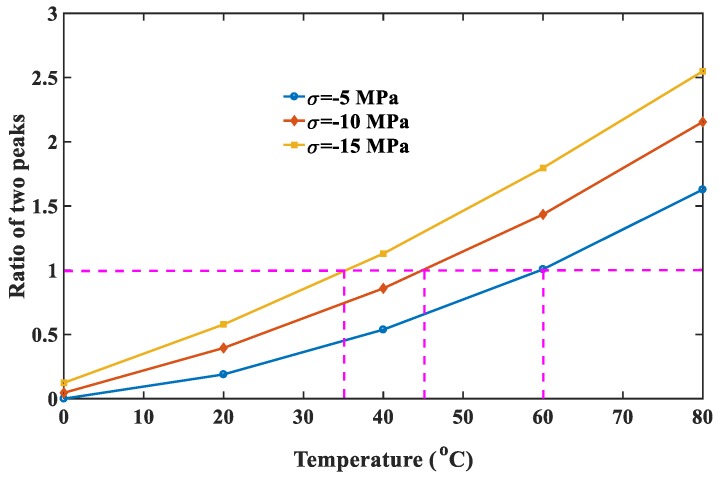
The ratio of the first peak to the second peak varied with temperature under different pre-stresses.

From the perspective of application, when ME devices using multiferroic nanocomposites operates in space stations or satellites where the temperature difference is large between day and night, the thermal stability of the ME effect is a critical issue for the performance and sensitivity of sensors. In the following discussions, the flexoelectric coefficient and intrinsic length are fixed at $\begin{array}{cc} 3\times 10^{-9} & C/m \end{array}$ and 1 nm, respectively. Figure 10 shows that the ME coefficient varied with magnetic field under different temperature increments. The negative ME coefficient under low magnetic field increases with increasing temperature, but the positive one under high magnetic field decreases with increasing temperature. For this reason, the first peak at the required field $H_{r1}$ can be improved by increasing temperature increment, while the second peak at $H_{r2}$ can be improved by reducing temperature increment or increasing temperature reduction. It is worth noting that the second peak in Figure 10 has the same variation tendency as the maximum value of the ME coefficient observed in previous experiments [42]. However, since the experimental study only focused on temperature effects of ferroelectric/ferromagnetic bilayer composites, which cannot obtain a dual-peak phenomenon in the ME coefficient versus magnetic field curve. Figure 11 shows that the peak values of the ME coefficient varied with temperature. It can be seen that the first peak surpasses the second one when the temperature increment is larger than 60 °C, which means one can reduce bias field under large temperatures to improve conversion efficiency of ME sensors using the first peaks. In practical applications, when the ambient temperature is fixed, an appropriate magnetic field should be applied. The other scenario is that temperature should be adjusted to obtain the maximum value of the ME coefficient when the applied field is given. In this case, the above-mentioned semiconductor refrigeration technology may be a perfect candidate, which can maintain the temperature at an optimal value to achieve the best performance of ME sensors. Another important feature observed in Figure 11 is that the relationship between peak values of the ME coefficient and temperature is linear, which can offer much convenience for the experimental design of multiferric devices. To clearly show the competitions between two peaks of the ME coefficient under different conditions, we introduce a dimensionless parameter η to represent the ratio of the first peak to the second peak. Thereupon, η can be used to determine the best performance of ME sensors under a certain condition. Specifically, η>1 means the first peak is the maximum value of the ME coefficient, and the applied field should be reduced to the required field $H_{r1}$; η<1 means the second peak is the maximum value, and the applied field should be enhanced to the required field $H_{r2}$. Figure 12 shows the variation of η with varying temperature under different pre-stresses. It indicates that a larger stress leads to a larger η. The critical temperatures when the first peak exceeds the second one are respectively 35 °C, 45 °C and 60 °C at −5 MPa, −10 MPa and −15 MPa.

**Figure 13:**
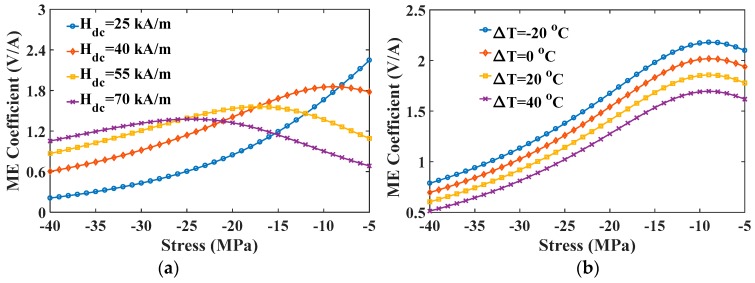
The ME coefficient varied with stress under different (**a**) magnetic fields and (**b**) temperatures.

The ME coefficient versus stress curve is plotted in Figure 13. In Figure 13a, for sensing a low magnetic field (25 kA/m in the figure), the value of the ME coefficient increases monotonically with decreasing the compressive stress. However, the variation trend for the case of a larger field has been changed. The ME coefficient shows a decreasing trend under a low stress and the curve shifts to the left overall with an increase in magnetic field. Consequently, as the static magnetic field increases, the optimal value of compressive stress corresponding to maximum ME coefficient increases. For example, the optimal value of compressive stress is −5 MPa at 25 kA/m, but 25 MPa at 55 kA/m. This result provides an effective method to obtain the maximum ME coefficient at fixed fields with an appropriate compressive stress. Figure 13b shows that temperature just influences the value of ME coefficient rather than the curve trend. The maximum value of positive ME coefficient at any stress can be obtained by reducing temperature.

## 5. Conclusions

In summary, a nonlinear multi-field coupling model for the ME effect in multiferroic nanocomposites is established, in which nonlinear characteristics of magneto-elastic-thermal coupling inherent to FM materials as well as size-dependent behavior of the component materials are considered. This model adequately predicated the influence of surface effect, flexoelectricity and multi-physical field loadings on ME coupling in multiferroic nanocomposites. The specific findings in this investigation may be summarized as:

(i) For the multiferroic composites which is thicker than a critical thickness (about 1 μm), the influence of surface layer and flexoelectricity cannot be negligible in accurately evaluating its ME performance. There may be ways and means of increasing the flexoelectric coefficient, or decreasing thickness, both of which can increase the value of the ME coefficient.
(ii) The strain gradient in multiferroic composites depends strongly on the thickness, and is influenced by external multi-filed conditions. A medium magnetic field could improve strain gradients. Besides, applying a large compressive stress or reducing temperature increments in the range of low magnetic fields will be beneficial for improving the strain gradient. However, the corresponding opposite operations for stress and temperature should be performed under a high magnetic field.
(iii) Dual-peak phenomena can be obtained in the ME coefficient of multiferroic nanostructures consisting of different FM materials. One can enhance a positive ME coefficient by increasing the flexoelectric coefficient or decreasing thickness, and optimize a negative ME coefficient by eliminating flexoelectricity.
(iv) Multiferroic nanocomposites operating under multi-field conditions exhibit significant multi-field coupling characteristics. Appropriate compressive stress and temperature can improve the ME coefficient at a fixed bias magnetic field. In particular, large compressive stress or temperature increments promotes the advantage of the first peaks in the ME coefficient curves, thereby reducing the required magnetic field.

The theoretical model provides a basic understanding of size-dependent behavior, and nonlinear characteristics of magneto-elastic-thermal coupling of nanostructured multiferroic composites. To some extent, it is useful for the optimized design of nano-devices operating in complex environments.

## Figures and Tables

**Table 1:** Material constants of FE and FM materials.

Constants	Terfenol-D	Ni	PZT
$E_{s}$ (GPa)	110	216	—
$\lambda_{s}$ (ppm)	1000	−37	—
$\mu_{0}M_{s}$ (T)	0.8	0.6	—
$\sigma_{s}$ (MPa)	200	−225	—
$\chi_{m}$	20	39	—
β (°C^−1^)	12 × 10^−6^	13 × 10^−6^	2 × 10^−6^
$s_{11}$ (m^2^/N)	—	—	14.8 × 10^−12^
$d_{31}$ (m V^−1^)	—	—	−175 × 10^−12^
$\varepsilon_{33}$ (C N^−1^ m^−2^)	—	—	1.55 × 10^−8^

## Appendix A

The detailed expressions of the equation set with respect to $\varepsilon_{10}^{m_{1}}$ and ρ are given in this section.

(A-1) $$\left[\begin{array}{cc} A_{11} & A_{12}\\ A_{21} & A_{22} \end{array}\right]\left[\begin{array}{l} \frac{1}{2\rho }\\ \varepsilon_{10}^{m_{1}} \end{array}\right]=\left[\begin{array}{l} B_{1}\\ B_{2} \end{array}\right]$$

where

(A-2) $$\begin{array}{l} A_{ij}=A_{ij}^{b}+A_{ij}^{s}+A_{ij}^{g}\\ B_{i}=B_{i}^{b}+B_{i}^{s}+B_{i}^{g} \end{array}$$

in which the right superscript b, s and g denote the terms related to the bulk layer, surface layer and strain gradient, respectively, and the detailed expressions of $A_{ij}$ and $B_{i}$ are

(A-3a) A11b=ε33tps11pε33−d312(tm1+tp)+tm2s11m2tA11s=1s11m2s(tp+tm1+2tm2)−tm1s11m1sA11g=2d31μ3113tps11pε33−d312

(A-3b) A12b=tm1s11m1+ε33tps11pε33−d312+tm2s11m2A12s=1s11m1s+1s11m2s

(A-3c) A21b=16(ε33tp3s11pε33−d312+tm13s11m1+tm23s11m2)+12ε33tp(tp+tm1)2s11pε33−d312+tm2s11m2(tp+tm2+tm12)tA21s=12tm12s11m1s+1s11m2s(tp+tm2+tm12)(tp+tm1+2tm2)A21g=2s11pε33s11pε33−d312μ31132ε33tp

(A-3d) A22b=ε33tp(tp+tm1)2(s11pε33−d312)+tm2s11m2(tp+tm2+tm12)A22s=1s11m2s(tp+tm2+tm12)−tm12s11m1sA22g=d31μ3113tps11pε33−d312

(A-3e) B1b=[(q11m1tm1s11m1+q11m2tm2s11m2)H1+(βm1tm1s11m1+βm2tm2s11m2)ΔT−ε33tps11pε33−d312(d31ppε33−βp)ΔT]B1s=(q11m1ss11m1s+q11m2ss11m2s)H1+(βm1ss11m1s+βm2ss11m2s)ΔT

(A-3f) B2b=tm2s11m2(tp+tm2+tm12)(q11m2H1+βm2ΔT)−12ε33tp(tp+tm1)s11pε33−d312(d31ppε33−βp)ΔTB2s=1s11m2s(tp+tm2+tm12)(q11m2sH1+βm2sΔT)−12tm1s11m1s(q11m1sH1+βm1sΔT)B2g=μ3113tps11pε33−d312(s11ppp−d31βp)ΔT

## Appendix B

**Nomenclature**			
Bks	Surface magnetic induction tensor	pip	Pyroelectric coefficient
C	Second-rank stiffness coefficient tensor	$q_{11}$	Equivalent piezomagnetic coefficient
$c_{ijkl}^{m}$	Equivalent elastic coefficient of FM phase	$s_{11}$	Equivalent compliance coefficient
cijklms	Surface elastic coefficient of FM phase	*Greek symbols*	
$c_{ijkl}^{p}$	Elastic coefficient of FE phase	$\alpha^{p}$	Thermal expansion coefficient of FE phase
cijklps	Surface elastic coefficient of FE phase	αps	Surface thermal expansion coefficient
$D_{l}$	Electric displacement vector	$\beta^{m}$	Thermal expansion coefficient of FM phase
Dls	Surface electric displacement vector	σ	Stress tensor
$d_{31}$	Piezoelectric coefficient	$\sigma_{ij}$	Stress tensor
$E_{k}$	Electric field vector	σijs	Surface stress tensor
$E_{s}$	Saturated Young’s modulus	$\sigma_{s}$	Saturation stress
Ejs	Surface electric field vector	$\tau_{ijk}$	Higher order stress
$e_{ijk}^{p}$	Piezoelectric coefficient	ε	Strain tensor
eijkps	Surface piezoelectric coefficient	$\varepsilon_{10}$	Centroidal strain
$g_{kij}^{m}$	Equivalent piezomagnetic coefficient	$\varepsilon_{33}$	Relative dielectric constant
gkijms	Surface piezomagnetic coefficient	$\varepsilon_{kl}^{s}$	Surface strain tensor
H	First-rank magnetic field tensor	$\lambda_{s}$	Saturated magnetostriction
Hks	Surface magnetic field tensor	$\chi_{m}$	Linear magnetic susceptibility
I	Second-rank unit tensor	$\mu_{0}$	Vacuum permeability
k	Relaxation factor	$\mu_{ijkl}^{p}$	Fexoelectric coefficient
$l_{s}$	Material intrinsic length	$\mu_{ki}^{m}$	Equivalent magnetic permeability
M	First-rank magnetization tensor	μkims	Surface magnetic permeability
$M_{s}$	Saturated magnetization		
